# Folic acid and neural tube defects: Discovery, debate and the need for policy change

**DOI:** 10.1177/09691413221102321

**Published:** 2022-06-23

**Authors:** Nicholas J Wald

**Affiliations:** 1 Professor of Preventive Medicine, Institute of Health Informatics, 4919 University College London, London, UK

**Keywords:** neural tube defects, folic acid, prevention, public health policy

## Abstract

Neural tube defects (NTDs) are a group of relatively common fatal or severely disabling birth defects that result in about 300,000 cases a year world-wide. The search for a cause was elusive, but in 1991 it was shown that about 8 out of 10 cases are due to a lack of vitamin B9 (folate) and are therefore preventable. This article (i) describes the challenge in finding the cause; (ii) examines the reasons for the failure of many countries to introduce folic acid fortification of staple foods such as flour and rice; (iii) shows that countries that have introduced fortification failed to do so in a fully effective way; (iv) shows how current preventive polices are confusing, inconsistent and sub-optimal; (v) shows that the proposed UK folic acid fortification policy is expected to prevent about 1 out of 10 NTD cases only; and (vi) proposes a simple, fully effective fortification policy that would prevent about 8 out of 10 NTDs and avoid the need for women to start taking folic acid supplements before pregnancy, a policy that has been shown to fail because only a small percentage of women adopt this practice.

## Neural tube defects and the search for a cause

This article describes a journey of discovery interrupted by delays and distracting debates that culminated in a global missed opportunity that, if fully realised, could have saved the lives and livelihoods of hundreds of thousands of individuals. But the journey is not over yet. There is further to go. Probably no-one could have predicted that a serious birth abnormality that occurs throughout the world in both wealthy and poor countries, amounting to about 300,000 cases a year, is caused by a vitamin deficiency. But this has proved to be the case. The abnormality is a neural tube defect (NTD), most commonly spina bifida or anencephaly, now known to be mainly caused by a deficiency of vitamin B9 (folate: see Box 1). The defects cause miscarriages, stillbirths and neonatal deaths, and they often lead to a termination of pregnancy if an NTD is identified through antenatal screening. Individuals born with spina bifida have disabilities that include paraplegia, incontinence of urine or faeces or both, and hydrocephalus.

**Box 1. Folic acid, folate and vitamin B9**

Folic acid is the ‘core’ molecule in all folates but is not itself present in natural food. Importantly, folic acid is more stable than natural folate and if added to food is not destroyed in cooking or heating as is the case with natural folate. The term “folate” is usually taken to include natural folate and synthetic folic acid. Recently, vitamin B9 has been used for ‘folate’ and sometimes as ‘vitamin B9 fortification’ when referring to folic acid fortification and as ‘vitamin B9 supplements’ when referring to folic acid supplements.

NTDs occur throughout the world with a wide variation in prevalence, ranging from about five per 10,000 to a hundred per 10,000. The search for causes was elusive. Migrant studies indicated that the NTD risk tended to move with the migrants; for example, the Irish migration to America resulted in Irish Americans having a high NTD risk but not as high as those who did not leave Ireland – evidence indicating an environmental factor.^1^ But what was the factor? Various theories were put forward, including a toxin from blighted potatoes that formed much of the diet among poor families in Ireland.^2^

A clue to finding what we now know as the main cause of NTDs arose from the work of a pathologist, Elizabeth Hibbard, and a paediatrician, Richard Smithells, in 1965 while working in Liverpool. Their work was prompted by a previous study showing severe folate deficiency in rat embryos either resulting in fetal death or deformity in almost all cases. They used a test first described a few years earlier called the FIGLU (formiminoglutamic acid) test.^3^ This involved the oral administration of the amino acid histidine, which is metabolised to glutamic acid. FIGLU is an intermediate in this reaction, which if impaired leads to an accumulation of FIGLU in the blood and excretion in the urine. Positive FIGLU tests can occur in various situations including folate deficiency. Hibbard and Smithells found that the FIGLU test was more often positive in NTD pregnancies than in unaffected controls, although the association was not specific; pregnancies associated with other abnormalities were also associated with positive FIGLU tests. However, the seed was planted.

Later, in 1983, Smithells and his colleagues found that women with a past history of an NTD pregnancy who took a multivitamin supplement before pregnancy and in the early stages of a subsequent pregnancy had a lower risk of having an NTD pregnancy than women with a similar history but who had not taken vitamin supplement.^4^ As the supplement contained several vitamins, if there were a genuine protective effect of one or more of them, it was not possible to identify which was the responsible vitamin or vitamins. Also, the study was not a randomised trial, and there was evidence of selection bias: women who took the multivitamin supplement tended to be of higher socio-economic status than women who did not. Therefore, at least in part, the lower risk in women who took the supplement simply identified a more affluent group that, anyway, had a low risk of having an NTD pregnancy.

In 1981, two years before Smithells reported his study,^5^ Michael Laurence and his colleagues in Cardiff conducted a randomised trial of folic acid (4mg/day), but unfortunately the trial was based on only 111 women, and the results, while consistent with a protective effect, were too small to provide an answer; there were two NTD pregnancies among the women allocated to the folic acid arm of the trial and four among the women allocated to the placebo arm of the trial.

In 1983, therefore, the scientific position was uncertain. The importance of the issue prompted me to seek support from the MRC to conduct a randomised trial that would be large enough to be informative and, by design, free of bias. The need for such a study was clear.^6^ The Medical Research Council reviewed a grant application and agreed to fund a trial for which I was the principal investigator. The trial became known as the MRC Vitamin Study.

## A distracting debate

One might have thought with MRC funding it would be plain sailing. Sadly, this was not the case.^7^ The principal investigator was accused by some of carrying out a useless study because it was most unlikely that simple vitamin deficiency could cause NTDs, and by others because the mere possibility that taking extra vitamins could prevent NTDs was enough to determine policy. Thus it was considered on the one hand a useless endeavour because extra vitamins could not work, and on the other hand, unnecessary research because they *might* work! Either way the study was stated by commentators in both camps to be unethical. Politics and personal opinions were hijacking the study. The issue was argued in the press and in Parliament. The heat of the debate led, at one point, to the MRC withholding funds for the study, a drastic set-back that was bravely salvaged by Sir James Gowans, head of the MRC at the time.

Several lessons can be learned from what happened. First, it is wrong to regard the scientific issues and the ethical issues as separate. The ethical issue depends on the interpretation of the science; if an intervention has been shown to be effective, it is unethical to conduct further research to see if that intervention is effective. If the scientific position is uncertain, it is both scientifically and ethically right to resolve the uncertainty and find out whether the intervention is effective. In the 1980s the question of whether any vitamin prevented NTDs was in the second category - uncertain.^6^

A second lesson to be learned is that politics and science make uncomfortable bedfellows. When there is scientific uncertainty over a medical issue that has captured public attention, politicians are under pressure to act. The science should not be manipulated to justify a particular political agenda.

A third lesson was the importance of randomisation, size of trial and scientific collaboration. Randomisation avoids bias, due for example to healthier women receiving the vitamins while less healthy women do not. Size of study is important to avoid results that could look favourable (or harmful) simply on the play of chance. Collaboration, often with colleagues throughout the world, helps achieve the study size needed, and also the wide “ownership” of the study across the globe serves an educational role, so that the results are more likely to be accepted and, potentially, put into practice.

## The MRC Vitamin Study

The MRC Vitamin Study,^8^ published in 1991, over 30 years ago, used a design that could reliably determine whether there was an NTD protective effect of any of the vitamins used in the study performed by Smithells and his colleagues, and if so whether it was folic acid or one of the other vitamins. The study included 17 UK centres, three in Australia, seven in Hungary, one in Israel, three in Canada, one in France and one in the USSR. Women with a history of an NTD pregnancy who were likely to receive preconceptual care were invited to join the trial. The dose of folic acid used in the trial was 4mg daily, the same as the dose used in the Cardiff trial and ten times the daily dose used in the study by Smithells and his colleagues.

The results of the trial^8^ were so clear-cut that the trial was stopped early. Among women allocated to receive capsules containing folic acid, the NTD rate was much reduced, by 71%, compared to the rate among women allocated capsules without the vitamin. In an “on-treatment” analysis limited to women who started taking their capsules before conceiving and continued doing so for several weeks into pregnancy, the risk reduction was 83%. There was no indication that any of the vitamins other than folic acid conferred an NTD protective effect.

The scientific uncertainty was resolved by the results of the MRC Vitamin Study. The study showed conclusively that an NTD was a folate deficiency disorder. In 1992 a Hungarian study^9^ was reported that used a multi-vitamin capsule supplement that included the vitamin folic acid at a daily dose of 0.8mg. There were only six NTD pregnancies in the study and the results could not be linked to folic acid specifically. The study was therefore inconclusive on its own, but the result was consistent with the MRC Vitamin Study result.

## The mixed public health response

Following the publication of the MRC Vitamin Study, public health authorities throughout the world, led by the USA and UK, acted promptly in advising all women who could become pregnant to start taking a folic acid supplement before they knew they were pregnant and to continue this for the first three months of pregnancy. This timing is important. In the embryo the neural tube closes about four weeks after conception. This means it closes about two weeks after the first missed menstrual period, or within six weeks from the first day of the last menstrual period. Starting to take folic acid supplements after a pregnancy has been confirmed is therefore invariably too late to be effective in preventing NTDs. Although the advice on when to start taking a folic acid supplement (before pregnancy and very early pregnancy) was sound, there was uncertainty over the dose of the vitamin.

The dose issue was conflated by access obstacles that disturbingly remain today. In various countries, including the UK and USA, a doctor's prescription is needed for folic acid supplements above 0.4mg (though 0.8mg supplements are available in health food stores). In other countries such as New Zealand, 5mg folic acid tablets are available without a prescription. In many countries such as the UK and USA 0.4mg daily is recommended for women who have not previously had an NTD pregnancy, but a higher dose, 4mg (or 5mg), is recommended for women who have had an NTD pregnancy. The UK also has a range of additional conditions in the 5mg/day category, including a family history of an NTD pregnancy in either partner, diabetes, sickle cell anaemia, treatment with anti-epileptic drugs and being a smoker.

The two-dose policy is illogical as the higher dose has been shown to be more effective.^10^ The dose-response relationship between folic acid intake and NTD reduction involves two steps. First the relation between folic acid intake and plasma folate is additive so that a unit increase in folic acid intake increases serum folate by a given amount.^10^ Second the NTD risk is inversely proportional to plasma folate, so that a percentage increase in plasma folate decreases NTD risk by a given percentage – approximately doubling the plasma folate approximately halves NTD risk.^10,11^ An analysis that combines both steps mathematically into one has been referred to as ‘teleoanalysis’.^11^ The main graph in the Figure shows the dose-response relation between folic acid intake and NTD prevention if the background serum folate is 5ng/ml, typical of many populations. The data in the two graphs below were used to derive the dose-response model based on folic acid intakes up to 1mg/day and serum folate levels up to 10ng/ml. As can be seen from the main graph, the model accurately predicts the NTD preventive effect of 4mg/day folic acid observed in the MRC Vitamin Study, namely 83%, a powerful test of the validity of the dose-response model.

**Figure.**
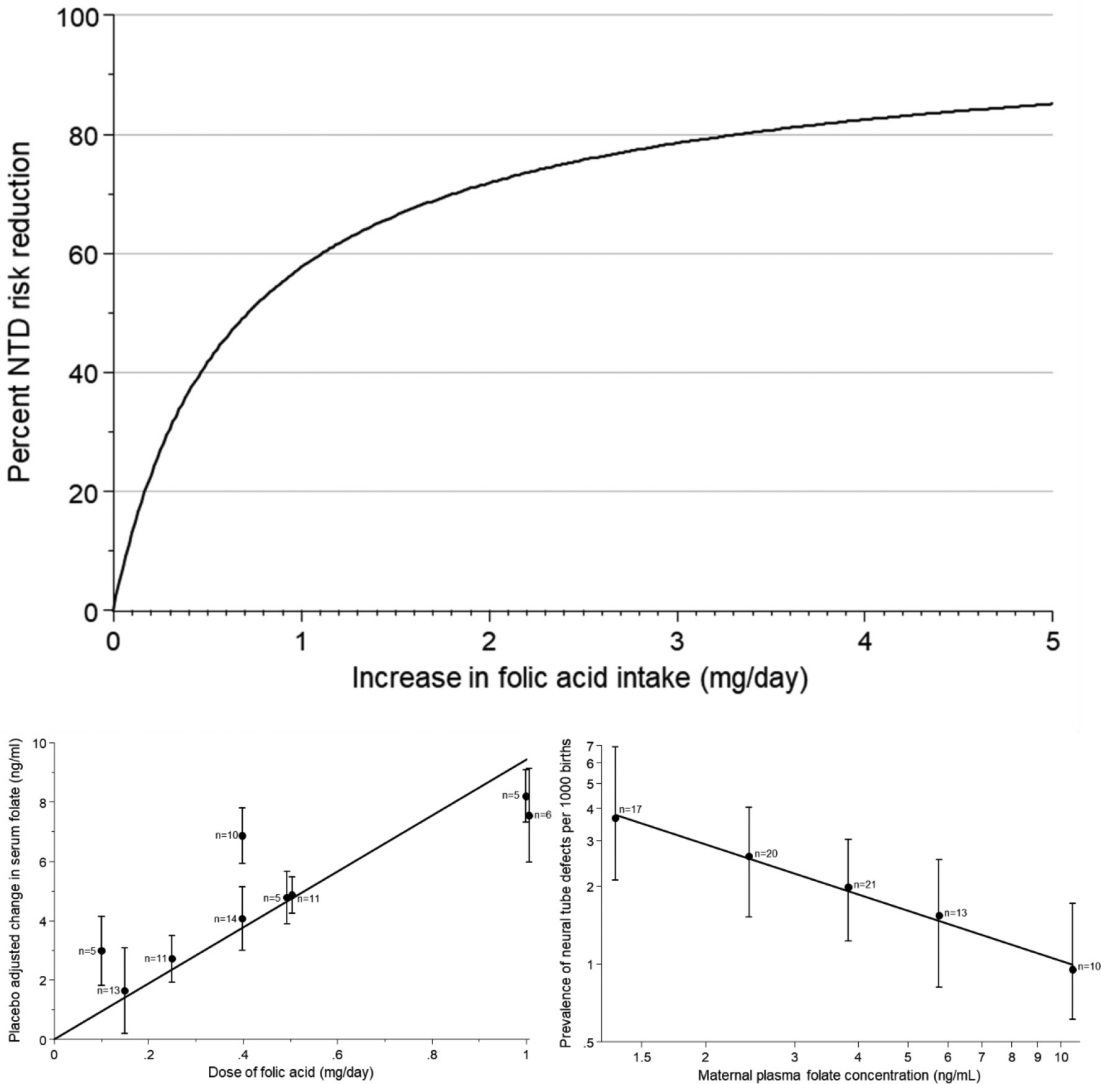
Main graph: percentage neural tube defect (NTD) risk reduction according to increase in folic acid intake per day for people aged 20–35 with a starting folate level of 5ng/ml. This is based on the equation: Percent NTD risk reduction = 100 × [1-[[[9.4 × increase in folic acid intake (mg/day) + 5]/5]^−0.81^]] derived from the data in the lower graphs, reproduced from Wald et al.^10^

The advice should have been to recommend 4mg/day for all women who could become pregnant. The regulatory authorities such as the Medicines and Healthcare Products Regulatory Agency (MHRA) in the UK and the Food and Drug Administration (FDA) in the USA should revise the advice given. They should also revise the regulations to allow 4mg (or 5mg) folic acid tablets to be sold without requiring a medical prescription since these doses are more effective and are non-toxic. Standardising the dose to 4 or 5mg daily remains an essential policy improvement that needs to be implemented. Limiting over-the-counter purchases of folic acid pills to 0.4mg was, as described below, based on a misinterpretation of historical data that incorrectly suggested that a high dose of folic acid might be neurotoxic. Furthermore, the graph in the Figure shows clearly that a 0.4mg per day dose has a relatively small impact on NTD prevention.

Whatever the supplement dose, it is not an effective NTD prevention policy because only a minority of women take a folic acid supplement before pregnancy. In a survey of nearly half a million women in England only 1 in 3 did so,^12^ and as few as 1 in 5 Afro-Caribbean and Asian women. Only 1 in 2 women who had a previous NTD pregnancy took a folic acid supplement before pregnancy. The policy of advising women to take supplements before pregnancy is a public health failure; an adequate level of folic acid fortification of staple foods is needed.

## Folic acid fortification

The substantial benefit of periconceptual folic acid supplementation shown by the MRC Vitamin Study in 1991 raised the issue of whether one or more staple foods should be fortified with folic acid. The concept of food fortification was not new. In the UK, for more than half a century, white wheat flour has been fortified with two other B vitamins (thiamine and niacin) and two minerals (calcium and iron). It was clear from the outset that a large proportion of pregnancies are unplanned and unexpected. The only sure way of ensuring a total population improvement in folate nutrition was through folic acid fortification. The United States mandated folic acid fortification of cereal grain products in 1996, which was fully implemented in 1998. The initiative was largely due to the efforts of a US Public Health Service doctor, Godfrey Oakley, and a paediatrician who was Medical Director of the March of Dimes, Dick Johnston.

Currently about 80 countries have adopted folic acid fortification. In all territories where fortification was introduced and monitored there was a reduction in the rate of babies being born with an NTD. No ill effects from fortification have been identified and nowhere has fortification been discontinued. Once a decision to fortify is reached the fortification level is critical. Chile in 2000 added 0.22mg folic acid per 100g wheat flour;^13^ the average folic acid intake increased by 0.43mg^13^ a day, with a decreased NTD risk of 43%,^14^ as was predicted from prior research.^10^ In 2009 theoretical concerns about safety led to Chile reducing the fortification level to 0.18mg per 100g wheat flour and this resulted in a 10% increase in the NTD rate.^15^

One might be forgiven for assuming that all countries have adopted folic acid fortification. Tragically this has not been the case. No European country has introduced folic acid fortification for reasons that defy logic and reason. In the UK a Government Advisory Committee, COMA, recommended in 2000 the addition of 2.4mg folic acid to 100g non-wholemeal wheat flour.^16^ In 2021 the UK Government announced that it will introduce mandatory fortification of non-wholemeal wheat flour. It did not specify the fortification level or when the measure would take effect.

In 2005 Irwin Rosenberg, Professor of Nutrition at Tufts University in the USA, stated “Folic acid fortification may be the most important science-driven intervention in nutrition in public health in decades”.^17^ Public health committees have, almost without exception, concluded that folic acid fortification be adopted. One needs to ask why it has not happened in many countries, and why many countries that have introduced fortification have done so only after considerable delay and at a low fortification level. Why has the human cost of needless miscarriages, stillbirths, neonatal deaths, therapeutic abortions and serious disabilities been tolerated? The ‘reasons’ are spurious. Johnston in 2008 stated that “The question of whether to add more folic acid to fortified grains is too important to public health to remain unanswered.”^18^

## ‘Reasons’ for failure to introduce folic acid fortification

The failure to fortify is a tragedy, but examining the ‘reasons’ may provide lessons on how to avert such public health failures in the future. Five issues have held back fortification:

1. The possibility of direct harm
  Several suggestions of direct harm have been made but none are scientifically valid. For example, a possible risk of colon cancer was raised because of a temporary rise in incidence around the time of folic acid fortification in the USA – a temporal association.^19^ However a causal association can be firmly rejected because there was no corresponding increase in mortality from colon cancer.^20^ The association arose because of the concurrent introduction of colon cancer screening, which temporarily results in an increased incidence through early detection. The underlying problem is that speculation and suggestion are incorrectly perceived as evidence. This problem is made worse by an unjustified greater concern over possible adverse effects that may accompany a proven health benefit, than the justifiable concerns over the harm that will certainly be done by not adopting the benefit. This is falsely fuelled by referring to the need for a risk-benefit analysis in which the risk (harm) is hypothetical but trumps a proven benefit. It would be better to simply conduct a “risk assessment” in which one assesses, based on evidence not speculation, the risk of how adopting a specific action compares with the risk of not taking that action. Policy is distorted by adhering uncritically to a so-called “precautionary principle”, which may sound sensible but is often an excuse for doing nothing, as has been the case with folic acid fortification.
2. Indirect harm from incorrect diagnosis
  - ‘Masking’
  Folate deficiency and vitamin B12 deficiency both cause a macrocytic anaemia. Folic acid can, in large enough doses, correct the anaemia of vitamin B12 deficiency without having any effect on the neurological damage that can be caused by vitamin B12 deficiency. This effect of folic acid has misleadingly been referred to as “masking” vitamin B12 deficiency. If this were true, it might cause the delay or missing of a diagnosis of vitamin B12 deficiency with the possible consequence of irreversible neurological damage. But there is no evidence that this is true. This concern was considered in 1998 by the US Institute of Medicine (IOM)^21^ (see Box 2) but not judged harmful, presumably because the IOM recognised that anaemia was not needed to make a diagnosis of B12 deficiency.
  During the second half of the twentieth century analytical tests for blood folate and vitamin B12 were introduced, and synthetic vitamin B12 became readily available. It then became clear which patient with a macrocytic anaemia needed treatment with folic acid and which with vitamin B12. The introduction of specific vitamin B12 assays together with improvements in clinical care meant that the presence or absence of an anaemia was not necessary in making a diagnosis of vitamin B12 deficiency; an estimated 28% of patients with a vitamin B12-deficient neuropathy present without anaemia.^22^ Standard of care requires that the investigation of patients with suggestive neurological symptoms should include a serum vitamin B12 assay, whatever the haematological findings. Reliance on haematological screening for neuropathic vitamin B12 deficiency is no longer acceptable medical practice and poses a risk of medical negligence claims.
  Stated simply, the concept of ‘masking’ is historical and has no place in current medical practice.^23,24^
  (b) Neurotoxicity
  The IOM, however, did reach a conclusion that turned out to be incorrect.^23,24^ They made an error that incorrectly indicated that there was evidence suggesting that folic acid could exacerbate the neuropathy of vitamin B12 deficiency and recommended a safe upper limit of 1mg folic acid intake daily. The IOM analysed data from the 23 small-scale studies referred to in Box 2, conducted 70–80 years ago (see Box 3).
  The error of falsely attributing neurological toxicity to folic acid has been an obstacle to instituting folic acid fortification in many countries. Retaining this 1mg per day upper limit creates a conflict of policies – folic acid fortification will shift the distribution of folate (including folic acid) intake to a higher level so that some people will exceed the supposed 1mg per day upper limit. Trying to increase average folic acid intake without increasing the number of people consuming more than 1mg per day severely constrains the fortification level, resulting in inadequate fortification and a smaller NTD preventive effect; a much larger effect would be achieved without this unwarranted and disproven upper limit.^8,10,11^
  (c) No case for an unsafe folic acid intake
  With the concept of ‘masking’ obsolete and the rejection of folic acid being neurotoxic, *there is no basis for the view that folic acid intake should be separated into safe and unsafe categories*. All categories should be considered safe and public health policy should be revised accordingly. **Box 2. ‘Masking’**
    The concern of ‘masking’ is mentioned in haematology textbooks but without supporting evidence. In 1998 the Dietary Reference Intakes report^21^ produced by the Food and Nutrition Board of the Institute of Medicine (IOM) of the US National Academies of Science assembled the results of 23 small-scale observational studies (including 11 case reports) of patients with pernicious anaemia (B12 deficiency) who were incorrectly treated with folic acid instead of vitamin B12. The studies were conducted about 70–80 years ago when tests for vitamin B12 and folate deficiency had not been introduced and treatments of the deficiencies were in their infancy. Much of the research was even before vitamin B12 was isolated and available for treatment. At that time, the distinction between folate deficiency and vitamin B12 deficiency was not recognised. The macrocytic anaemia of either vitamin deficiency was treated with daily doses of 5mg or more of folic acid. Folic acid was isolated and available in synthetic form in 1945, three years before vitamin B12 was first isolated and available for therapy and three decades before it was synthesized in 1976. Patients with clinical vitamin B12 deficiency had been treated with the wrong vitamin. Though the macrocytic anaemia was corrected, the vitamin B12 deficiency remained untreated, with resulting neurological damage, including irreversible spinal cord damage. The IOM did not present a quantitative analysis but simply reported “in most cases throughout the dose range, folate supplementation maintained the patients in haematological remission over a considerable time span”^21^ without explicitly regarding it as harmful.
    **Box 3. Neurotoxicity**
    Using data from the 23 studies referred to in Box 2, the IOM noted that there were more than a hundred reported cases of neurological progression among the patients with vitamin B12 deficiency who received folic acid at doses of 5mg per day or greater, but only eight reported cases among patients receiving doses of less than 5mg per day. They concluded that a high dose of folic acid was potentially neurotoxic. The flaw in the IOM analysis is that the denominators were ignored: in the 23 studies twelve patients took a lower dose of folic acid and eight developed neuropathy, i.e. 67%, while 279 patients took the higher dose and 147 developed neuropathy, i.e. 53%. There was no evidence, therefore, that a high dose of folic acid was toxic to the nervous system. The flaw in the IOM analysis was identified in 2018^25^ and put into a historical context in 2019.^26^ The IOM went further; the possibility that 5mg a day or more was harmful prompted them to arbitrarily reduce this limit fivefold to one fifth, i.e. 1mg per day. This was labelled the tolerable upper intake level. This limit is unjustified because of the flawed analysis but has, in error, been widely adopted. It should be abandoned. The suggestion that folic acid is neurotoxic can be firmly rejected and should not affect policy.
3. Hypothetical harm versus proven benefit
  On the one hand it is an established fact that a low folic acid fortification level will be harmful in unnecessarily restricting the number of NTDs prevented – as many as about 1 per 1000 pregnancies. On the other hand there is only hypothetical speculation that neuropathic B12 deficiency might escape early clinical detection from an incidental finding of a macrocytic anaemia. Even assuming rare cases escape early clinical detection, the balance is heavily weighted in favour of implementing adequate folic acid fortification. A safe proven benefit should not be denied because of an unproven and hypothetical perception of a hazard that if existent, is rare. For example, in 2019 the UK Committee on Toxicity (COT) (https://cot.food.gov.uk/sites/default/files/cotfolicacidstatement.pdf) accepted that the IOM analysis was flawed and that folic acid was not neurotoxic. Nonetheless, without providing evidence, it adhered to the unwarranted opinion that ‘masking’ could result in failing to diagnose a vitamin B12 deficiency neuropathy in a timely manner.
  Recognising why this is not a justified or evidenced-based assessment is important in influencing a fully effective fortification policy. The COT report concludes that “It is well established that folic acid can delay the diagnosis of pernicious anaemia, allowing progression of the neurological damage until it is severe and potentially irreversible” (para 50). In spite of there being no evidence for this, the COT report retains the 1mg per day folic acid intake upper limit proposed by the IOM,^21^ a limit that was based on inappropriate and flawed analysis incorrectly indicating that folic acid was neurotoxic at high doses (see Box 3). A correct analysis indicates no neurotoxicity and no basis for a 1mg per day upper limit. The COT report seems to have applied this upper limit in respect of ‘masking’ even though its derivation by the IOM had nothing to do with ‘masking’. The COT report also does not consider the harm done in failing to prevent NTD pregnancies as a consequence of limiting folic acid intake. The COT report's endorsement of the 1mg per day folic acid intake upper limit is not evidence-based and should not be used to influence fortification policy.
  While these points challenging the conclusions of the UK COT report need to be considered by the UK Government, they may also help other governments overcome unwarranted barriers to implementing fully effective fortification. Four conclusions can be drawn from all the evidence: (i) folic acid is not neurotoxic, (ii) there is no basis for having a 1mg per day folic acid intake upper limit, (iii) there is no evidence for ‘masking’ being retained as a valid concept that causes harm, and (iv) failure to capture the benefit of folic acid intakes greater than 1mg per day causes harm.
  The COT report illustrates how a speculative and unlikely side-effect can unjustifiably outweigh a proven benefit.
4. Philosophical considerations
  What can be described as philosophical arguments against folic acid fortification hinge on three issues: it removes consumer choice, encourages a “nanny state”, increases vitamin B9 intake for members of the population that might not directly benefit (e.g. men).
  One can dismiss these arguments. The widespread addition of salt to processed foods over which consumers have little choice has enormous adverse health effects. It is a double standard to argue against folic acid fortification (that is beneficial) but tolerate the addition of salt to natural food (which is harmful). To its credit, in 2021 the UK Government announced that fluoride would be added to tap water to help reduce tooth decay, a public health strategy directly comparable to folic acid fortification.
  Governments need to make decisions that promote health and encourage the manufacture and processing of food and drinks in a safe and transparent manner so that consumers can know the composition of what they eat and drink. Much of public health involves collective decisions on the safety of buildings, roads, air and water, appliances, food and beverages. These are examples of actions taken by a civilised state. What is important is that the regulations are rational, transparent and based on evidence. The argument that only pregnant women benefit from fortification so why should the rest of the population be “exposed” to folic acid reflects a basic misunderstanding; fathers, families, society and the public purse are affected by miscarriages, terminations of pregnancy, stillbirths, neonatal deaths and children with severe disabilities, not solely pregnant women and mothers.
5. Mandatory or voluntary fortification
  After decades of experience globally the technology and logistics of fortification are well established. The cost of folic acid fortification is minimal. If all producers of grain products need to comply with national fortification standards, then there is “a level playing field” without one flour or grain miller fortifying and absorbing the minimal cost but not others; this is why, for example in the UK, the millers have supported mandatory fortification but have not to any material extent adopted voluntary fortification. They all make the reasonable point that it is the role of the Government to determine these health-focused regulations, not commercial companies to take on this responsibility. In this assessment, the full human and economic costs of failing to prevent as many NTDs as possible have too often been overlooked and ignored by advisory committees and policy-makers. It is simply wrong to ignore them and the adverse impact they have on individuals, families, the health system, schools and social service agencies.
  Box 4 summarises four principles that arise from the analysis of the above five issues and could help guide fortification policy.

**Box 4. Principles relating to folic acid fortification**

(i) There is no reason to limit folic acid intake.
(ii) It is a mistake to separate folic acid intakes into safe and unsafe categories. All intakes that have been used in medical practice have been shown to be safe. This means abolishing the 1mg upper limit which should no longer be an issue of concern.
(iii) The objective in fortification should be to add sufficient folic acid to achieve the full achievable effect in preventing NTDs.
(iv) It is for Governments to mandate fortification as a legitimate public health measure.
(v) In any risk assessment all the evidence must be examined, but when there is human evidence available, as there is with folic acid, this should take precedence over other evidence (e.g. animal or toxicological), and speculation should not be accepted as evidence. The assessment must recognise that the withholding of a benefit is a harm and that the consequences of having a preventable NTD pregnancy, stillbirth or a child with spina bifida is costly in both human and financial terms.

## What to fortify and by how much

Flour and grains, such as rice, are sensible ‘vehicles’ for fortification because they are part of people's daily diet without wide variation in intake. Also, the supply and milling of grains is, in many countries, industrialised among relatively few companies, making fortification highly efficient.

A problem, however, is that fortification – where introduced – has in many countries been limited to white bread flour, which perversely means that people choosing healthier whole grain bread are denied the health benefit of folic acid fortification. In many countries fortification is limited to wheat flour which means that people with gluten intolerance are denied the benefit even though this affects about 6% of the population. Families that tend to eat rice instead of bread will also be denied the benefit in countries that do not fortify rice. This overly selective application of what to fortify creates inequity in the delivery of a public health measure. The restrictions are confusing and unfair. They tend to favour certain ethnic and racial groups over others. Consumer choice could be accommodated by requiring unfortified flour or other grain products to be labelled, for example, as “Not fortified with folic acid (vitamin B9)”. All flour and grain products should be fortified with folic acid unless so labelled. In countries where flour milling is not centralised it may be necessary to select a non-flour vehicle for fortification or even several vehicles to avoid exacerbating health inequalities.

The fortification level should be sufficient to safely achieve the full preventive effect. This, in turn, means understanding the dose-response relationships between folic acid intake and NTD prevention (See Figure).^10,11^ Folic acid fortification must be optimal not partial. Evidence shows that this can be achieved with a fortification level that results in an average daily folic acid intake of 4mg a day – the amount used in the MRC Vitamin Study.

Folic acid fortification that raises average plasma folate levels from baseline levels of about 5ng/ml to, say, 20ng/ml would prevent about 40–50% of NTD cases. It would be about 80% if the level were about 40ng/ml, which is achieved with 4mg/day folic acid. The UK government is currently considering the fortification of non-wholemeal bread flour only, at a fortification level of 0.2mg per 100g flour, which they estimate will prevent just 8–12% of NTDs (https://assets.publishing.service.gov.uk/government/uploads/system/uploads/attachment_data/file/808698/folic-acid-impact-assessment.pdf, p.15). Such a minimalist approach will come at a significant human cost. It could take decades for the UK to revisit the policy and enhance the scope and level of folic acid fortification needed to achieve substantial NTD prevention. However, the UK now has the opportunity to adopt a maximum NTD prevention policy from the outset and in doing so become a world leader; all countries that fortify flour or grain with folic acid do so at too low a level, and a UK example would encourage other countries to increase the level and scope of their mandated folic acid fortification.

## The global cost of the failure to fortify

It has been estimated that globally there are approximately 300,000^27^ babies born with an NTD or pregnancy terminations each year on account of an NTD, of which an estimated 240,000 could be prevented with a fortification policy that delivers 4mg folic acid daily. Between 1992 and 2020, folic acid fortification could have prevented 240,000 × 28, i.e. 6,720,000 NTD cases. Even if the fortification policy originally adopted by Chile had been followed, this would have prevented 120,000 × 28, i.e. 3,360,000 NTD cases. By comparison with the millions adversely affected by NTDs, the total cumulative number of people with severe limb-defect abnormalities caused by thalidomide is about 10,000 worldwide.^28^ Thalidomide was effectively banned because of the birth defect it caused, but the birth defects arising from vitamin B9 (folate) deficiency are tolerated even though there are about three hundred times more NTDs than thalidomide cases. Sadly, this illustrates how banning a harmful substance is regarded as more important than adding a beneficial substance when, of course, they should be treated as equally important.

## The case for fully effective fortification

The pressing issue is to avoid ‘token’ fortification that will achieve minimal NTD prevention and thus defeat the whole point of fortification. It should not be acceptable to adopt a policy designed to prevent 20% of NTDs when 80% could be safely prevented simply by increasing the fortification level and the range of staple foods that are fortified. Once the decision to fortify has been taken there is no practical or moral justification for rejecting a more effective version of fortification in favour of a less effective version.

There is a further ethical perspective. For over thirty years antenatal screening for NTDs has been routinely adopted throughout the world. In England and Wales, between 2007 and 2017, 4425 NTD cases were diagnosed in pregnancy and led to a termination with an increased trend over time._­_^29^ It is surely wrong to provide this service as an alternative to primary prevention, particularly when primary prevention through folic acid fortification can, at the appropriate fortification level, be highly effective. Primary prevention spares families the distress of a positive screening result and the difficult decision of a pregnancy termination if the fetus is affected. Effective primary prevention is the priority, supported by antenatal screening. Women have routine antenatal ultrasound scans that would detect most of the NTD pregnancies not prevented by folic acid fortification.

Having acknowledged that primary prevention should be the priority, it is also necessary to acknowledge that the current position on NTD prevention is inconsistent and unsatisfactory. It relies on sub-optimal folic acid fortification that requires the continued use of periconceptual folic acid supplements (at one of two doses, depending on a woman's past medical history) even though it is known that most women fail to take supplements before their pregnancy. The supplement strategy has widened health inequalities because women in higher socioeconomic groups are more likely to take these supplements and do so before becoming pregnant. There is, therefore, an urgent need to use all the evidence available to specify a new strategy that will achieve the full NTD-preventable effect across the whole population.

Specifically, the action needed is to fortify at a level that would provide an average daily intake of 4mg folic acid, not 0.4mg as previously suggested.^30^ Taking peri-conceptional folic acid supplements would then no longer be needed. The whole population would benefit, avoiding inequalities with some women buying such supplements but most others not doing so. Box 5 shows how an extra 4mg/day folic acid intake converts to a fortification level of 1mg/100g flour or grain. The method avoids having to rely on estimating a person's average daily consumption of flour and grain which is imprecise and difficult to determine.

**Box 5. Setting the folic acid fortification level**

(i) An average added folic acid intake of 4mg per day needs to be converted to an amount, say Xmg per 100g flour or grain, and this is what needs to be specified in the appropriate regulations. X will depend on the average consumption of flour and grain in a given population. Estimating this is difficult and imprecise.
(ii) A pragmatic approach is needed. The serum folate level in the women in the MRC vitamin study who took 4mg/day folic acid was 44ng/ml and was 5ng/ml in the controls, i.e. a 39ng/ml increase. The 44ng/ml could be regarded as the target level. US fortification of 140μg/100g increased serum folate by 5.4ng/ml.^31^ To achieve a serum level of 44ng/ml, (140 x $\frac{39}{5.4}$ ) μg/100g i.e. 1mg/100g (10ppm) flour or grain would be needed.
(iii) Periodic monitoring of serum folate levels could be carried out to check on these levels in population samples; if the serum folate were more than 6ng/ml off target, the measurements would be repeated 1-2 months later and, if confirmed, adjusting the fortification level could be considered.

While some will say it is unrealistic to think that countries will fortify at a level of 1mg per 100g of flour or grain, advice should be based on the science without second-guessing what policy-makers will do. There are examples of what was regarded as unrealistic at one point in time but later became widely accepted with little or no recollection of the original opposition, such as vaccination or banning smoking in pubs. There is no doubt that perpetuating the false dichotomy between a daily folic acid intake that is currently acceptable (<1mg/day) and one that is currently unacceptable because it is too high (>1mg/day) is seriously restricting the opportunity to secure the full preventive impact of fortification.

All the evidence taken together shows that the full NTD-preventable effect would be achieved if folic acid fortification were set at a level and in a range of foods that would increase average folic acid intake by 4mg per day. This is the safe, inexpensive and equitable policy of choice. Unlike current policies, nearly all NTDs would be prevented. It is a policy that, at one stroke, would overcome the present confusion and the limited impact of current practice.
